# Loss of Par3 promotes lung adenocarcinoma metastasis through 14-3-3ζ protein

**DOI:** 10.18632/oncotarget.11728

**Published:** 2016-08-31

**Authors:** Song Tong, Tian Xia, Kai Fan, Ke Jiang, Wei Zhai, Jing-Song Li, Si-Hua Wang, Jian-Jun Wang

**Affiliations:** ^1^ Department of Thoracic Surgery, Union Hospital, Tongji Medical College, Huazhong University of Science and Technology, Wuhan, China

**Keywords:** NSCLC, Par3, 14-3-3ζ, metastasis

## Abstract

Partitioning defective protein 3 (Par3) can activate the Tiam1/Rac pathway to inhibit invasion and metastasis in many cancers; however, the role of Par3 in lung adenocarcinoma remains unknown. Here we show that Par3 is downregulated in lung adenocarcinoma tissues and is associated with higher rates of lymph node metastasis and recurrence. Our functional study demonstrated that knock-down of Par3 promoted lung adenocarcinoma cell growth, cell migration, tumor formation, and metastasis, all of which were effectively inhibited when 14-3-3ζ was silenced. We found that Par3 binded with 14-3-3ζ protein and also showed that Par3 abrogated the binding of 14-3-3ζ to Tiam1, which was responsible for Rac1 activation. Knock-down of 14-3-3ζ inhibited Tiam1/Rac-GTP activation and blocked the invasive behavior of cells lacking Par3. These data suggest that loss of Par3 promotes metastatic behavior in lung adenocarcinoma cells through 14-3-3ζ protein.

## INTRODUCTION

The vast majority of lung cancer cases can be categorized as non-small cell lung cancer (NSCLC), which includes, broadly, lung adenocarcinoma (LuAC), squamous cell carcinoma, and large cell carcinoma subtypes [1]. Metastasis is the leading cause of LuAC treatment failure and mortality [2]. Understanding the mechanisms of cancer metastasis is important for further development of novel therapeutic agents and for the treatment of metastatic NSCLC.

In mammals, the partitioning defective (Par) family of cell polarity proteins, including MARK kinases (Par1), Par3, Par4 (LKB1), Par5 (14-3-3), Par6 and protein kinase C ι/ζ (aPKC), plays key roles in many aspects of cells such as polarization, migration and proliferation [3]. The Par complex (Par3, Par6, aPKC, and Cdc42) regulates various processes such as the establishment of directional cell migration, apical-basal polarity and asymmetric cell division [4]. The Par complex member Par3 is a scaffold protein that contains three PDZ domains, an N-terminal domain, and a C-terminal domain. The PDZ domains bind with proteins such as Par6 and the phosphatase and tensin homologue (PTEN) [5, 6]. The N-terminal domain is important for apical localization and dimerization of Par3 [7]. The C-terminal domain binds with aPKC to inhibit its activity and with a Rac exchange factor Tiam1 (T lymphoma invasion and metastasis) to inhibit its exchange activity [8].

14-3-3 proteins have an important role in a wide range of biologic processes by binding to a variety of proteins. 14-3-3ζ, a member of the 14-3-3 protein family, acts as a oncogene in cancer and has a central role in tumor progression [9]. Previously study demonstrate 14-3-3ζ could binding to Par3 and disrupts the function of the Par3/Par6/aPKC polarity complex [10].

Par3 has been considered a suppressor of invasion and metastasis. Knock-down of Par3 causes Tiam1/Rac-GTP pathway activation and induces breast tumorigenesis and metastasis [8, 11]. However, other reports have shown that Par3 may have pro-oncogenic activity in skin cancer and squamous cell carcinomas [12]. The functions of Par3 in LuAC metastasis have not been investigated. Here, we present evidence that loss of Par3 promotes LuAC invasion and metastasis through 14-3-3ζ protein. Furthermore, our results show that Par3 abrogated the binding of 14-3-3ζ to Tiam1 that was responsible for Rac1 activation, presumably resulting in the inhibition of LuAC metastasis.

## RESULTS

### Par3 expression is frequently lost in human LuAC

**Figure 1:**
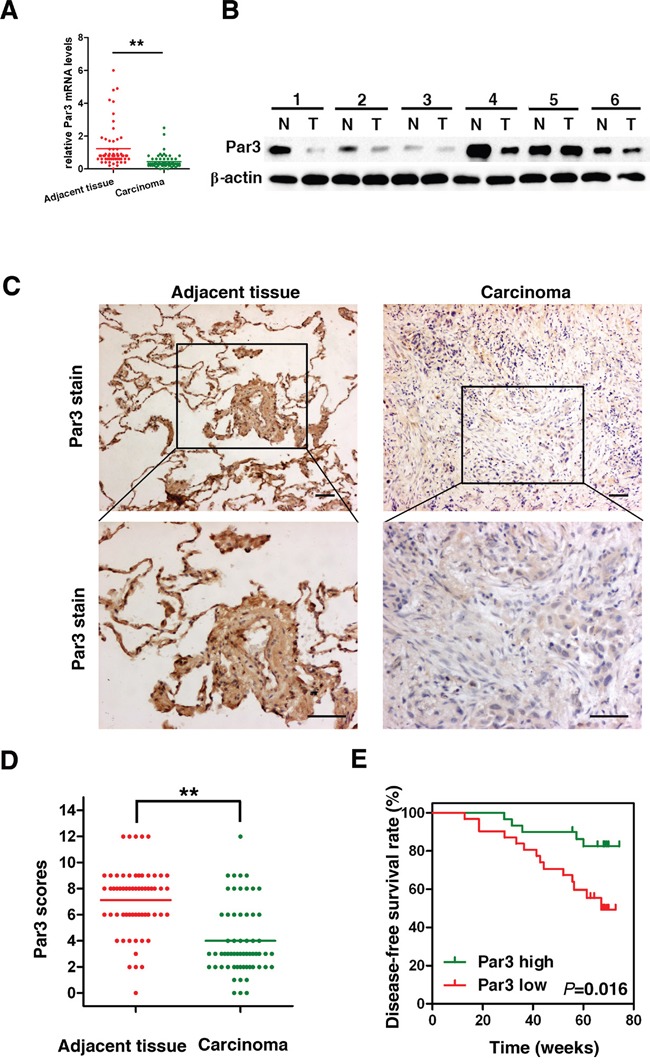
Par3 expression is frequently lost in human LuAC **A.** Relative expression levels of Par3 detected by RT-PCR in 61 pairs of LuAC tissues. **B.** Western blot analysis of Par3 expression in total frozen samples lysates originating from 12 human LuAC samples (T) and matched adjacent tissues (N). **C.** Representative immunohistochemical staining of Par3 expression in human LuAC and adjacent tissue. Bars: 100 μm. (original magnification, 100× and 200×) **D.** Par3 expression scores are shown as dot blots. LuAC samples and matched adjacent tissues were compared using paired Student's t-test. n = 61. **E.** Kaplan–Meier analysis indicating the correlation between the reduction in Par3 expression and poorer disease-free survival rates of LuAC patients. * *P* < 0.05, ** *P* < 0.01.

The expression of Par3 was studied by RT-PCR in 61 pairs of primary LuAC samples versus adjacent tissues. A significant reduction in Par3 gene expression was apparent in LuAC compared to normal lung tissue (Figure 1A, *P* < 0.01). Western blot analysis showed that Par3 was frequently under expressed in LuAC compared its expression in adjacent normal tissues (Figure 1B). We performed immunohistochemical analyses to examine the expression level of Par3 in 61 human LuAC specimens. As shown in Figure 1C, the immunostaining intensity of Par3 was significantly weaker in LuAC sections than in matched adjacent tissues. Quantification analyses further demonstrated that Par3 protein expression was remarkably reduced (Figures 1D, *P*< 0.01). The association of Par3 expression with clinicopathological features in 61 LuAC samples with informative IHC was statistically analyzed. The results showed that the reduction in Par3 expression was significantly associated with lymph node metastasis (*P* = 0.01) and recurrence (*P* = 0.04, Table 1). Furthermore, Kaplan-Meier analysis revealed that the reduction in Par3 expression was significantly associated with poorer disease-free survival (DFS) rates in LuAC patients (Figures 1E, *P*< 0.05). Multivariate Cox regression analysis showed that the hazard ratio (HR) for DFS with tumors with high Par3 expression was higher than that of tumors with low Par3 expression (HR 2.07 95% CI 1.12-3.83, *P* = 0.02).

**Table 1:** The association between clinical parameters with Par3 mRNA

*Features*	*No.*	*Par3*		P-*value*
		*Low*	*High*	
*Gender*				
*Male*	33	12	21	0.06
*Female*	28	18	10	
*Age*				
*≤60*	24	12	12	0.79
*>60*	36	18	18	
*Smoking status*				
*Nonsmokers*	39	15	25	0.002
*Smokers*	21	15	6	
*Tumor size*				
*≤3cm*	24	10	14	0.08
*>3cm*	37	20	17	
*Lymph node metastasis*				
*Without*	41	16	25	0.01
*With*	20	14	6	
*Stage*				
*I, II*	39	18	21	0.19
*III, IV*	22	12	10	
Recurrence*				
*No*	43	18	25	0.04
*Yes*	17	12	5	

* Partial data not available; statistics based on available data.

### Loss of Par3 increases tumor growth *in vitro and in vivo*

**Figure 2:**
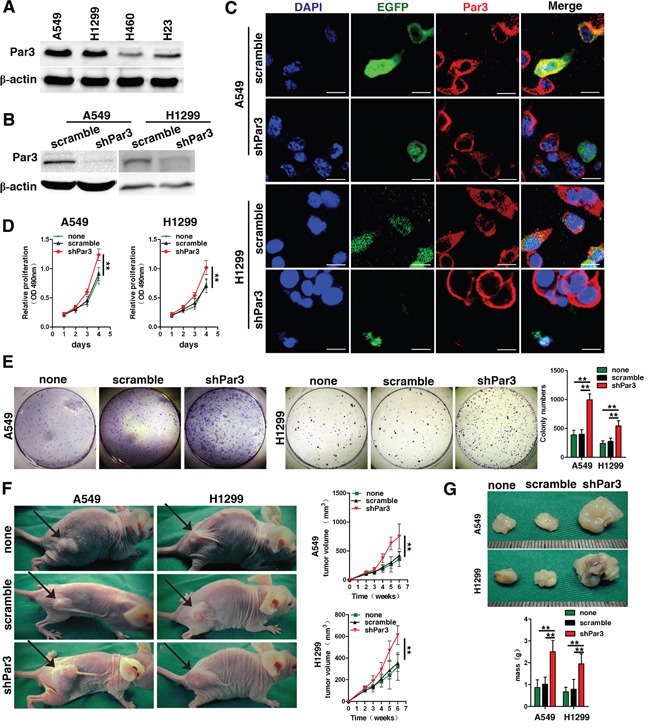
Loss of Par3 increases tumor growth **A.** Western blot analysis of Par3 expression in 4 LuAC cell lines. **B.** Validation of shRNA against Par3 in A549 and H1299 cells by Western blotting. **C.** Validation of shRNA against Par3 in A549 and H1299 cells by immunofluorescent staining. Bars: 25 μm. **D.** Growth curves between Par3-shRNA and scramble control-infected cells by MTT assay. The results are expressed as the mean±standard deviation (SD) of five independent experiments. **E.** Downregulation of Par3 significantly increased colony formation in A549 and H1299 cells. Quantitative data for colony numbers are shown in the right panel. **F-G**. Images of the xenografted tumors formed in nude mice injected with infected A549 and H1299 cells. The volumes and weights of tumors are shown in the down panel. There were 10 mice in each group. * *P* < 0.05, ** *P* < 0.01. none, Non infected cells.

We found that Par3 is highly expressed in A549 and H1299 cells, compared with H460 and H23 cells (Figures 2A). To assess the effect of the Par3 in LuAC, we used short hairpin RNAs (shRNAs) targeting Par3 in A549 and H1299 cells. Western blotting and immunofluorescence staining analyses were used to detect the changes in Par3 expression after infection (Figures 2B and 2C, supplement Figure 1 contain the negative staining control). The MTT assay showed that cell growth rates in shPar3-infected cells were significantly higher than those in the scramble control cells (Figures 2D, *P* < 0.01). The colony formation assay yielded a higher number of colonies as well as larger colonies in the shPar3-infected cells compared to the control cells (Figures 2E, *P* < 0.01). A549 subcutaneous tumors were established in the right dorsal flank of nude mice. Six weeks later, we found that downregulation of Par3 resulted in significantly accelerated growth of tumors (Figures 2F and 2G, *P* < 0.01,).

### Downregulation of Par3 promotes invasion of LuAC *in vitro*

**Figure 3:**
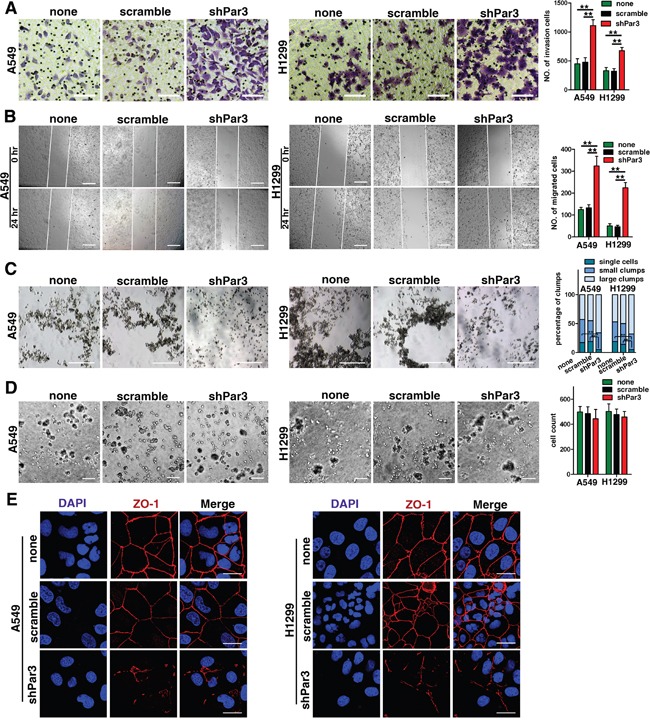
Downregulation of Par3 promotes invasion of LuAC *in vitro* **A.** Transwell invasion assay showing that downregulation of Par3 promoted cell motility and invasion. The numbers of invading cells are summarized in the right panel. The results are expressed as the mean±SD of five independent experiments. Bars: 100 μm. (magnification, 200×) **B.** Representative images from scratch wound migration assays indicated that loss of Par3 increased the migratory potential of A549 and H1299 cells. Bars: 100 μm. (magnification, 200×) **C.** Cells were cultured in hanging drops for 18 h, and representative images are shown. The clump size was categorized into single cells, small clumps (2–10 cells), and large clumps (>10 cells). The percentages of clumps are presented in a bar graph. The results are expressed as the mean±SD of five independent experiments. Bars: 100 μm. **D.** A phase contrast image shows that the number of cells adhering to the Matrigel did not differ between the two groups. Bars: 100 μm. All quantitative data are shown in the down panel. * *P* < 0.05, ** *P* < 0.01. **E.** Immunofluorescent staining of tight junction proteins localization (ZO-1) in different groups. Bars: 25 μm.

A Transwell invasion assay revealed significant increases in cell invasion and motility upon downregulation of Par3 (Figures 3A, *P* < 0.05). The scratch wound migration assays showed that shPar3 -infected group have more migrated cell compared with control group (Figures 3B, *P* < 0.05). To determine whether downregulation of Par3 affects cell-cell interactions, we performed a hanging-drop assay by suspending cells in drops of media hanging from the culture dish lid. Loss of Par3 in A549 and H1299 cells induced a decrease in the number of cell clumps, demonstrating decreased cell cohesiveness (Figures 3C, *P* < 0.01). On a Matrigel-coated surface, the adhesive ability of cells did not change in different groups (Figures 3D). We next examined the effect of Par3 on protein of tight junctions in A549 and H1299 cells. The disruption of ZO-1 localization in shPar3 -infected cells was particularly severe and clearly visible (Figures 3E).

### Loss of Par3 promotes LuAC tumor metastasis and angiogenesis

**Figure 4:**
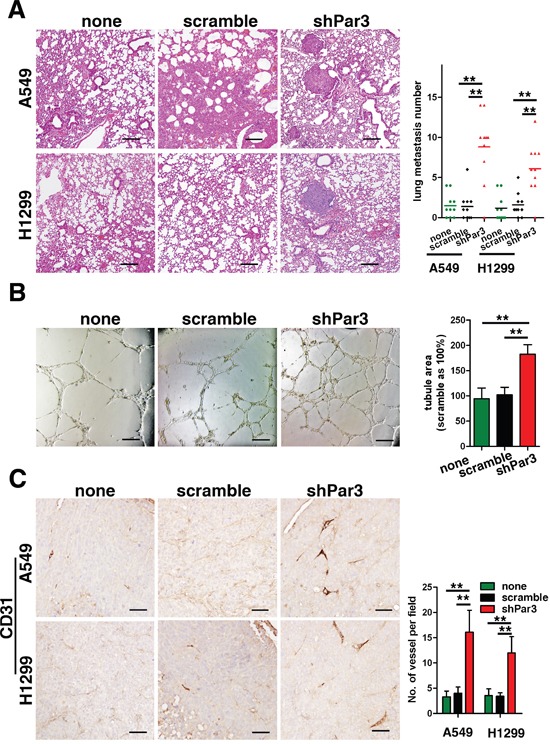
Loss of Par3 promotes LuAC tumor metastasis and angiogenesis **A.** In experimental metastasis assays, A549 cells were injected in the spleen and representative images of H&E-stained sections are shown for the lung. Bars: 100 μm. **B.** Loss of Par3 promotes the angiogenic potential of LuAC cells *in vitro*. HUVECs were infected with lentiviral carrying Par3 shRNA or control shRNA and cultured with Matrigel. After incubation, endothelial cell tube formation was assessed under phase-contrast microscopy, and the tube area was measured. Bars represent the mean±SD from triplicate experiments. Bars: 100 μm. **C.** Tumor angiogenesis was assessed with IHC staining using an antibody against CD31. Blood vessels were quantified in three sections (six random fields/section) from each mouse. Bars: 25 μm. All quantitative data are shown in the right panel. * *P* < 0.05, ** *P* < 0.01.

To investigate the effect of downregulation of Par3 on LuAC metastasis *in vivo*, we conducted an experimental metastasis assay by injecting A549 and H1299 cells into the spleen of nude mice. As shown in Figure 4A, H&E staining revealed that the number of lung micrometastatic nodes was significantly higher in the group injected with shPar3-infected cells (*P* < 0.01). This results is similar in previously breast cancer study [8]. Angiogenesis are important phenomena involved in the metastasis of cancer cells and they are associated with a poor prognosis [13]. To explore the role of Par3 on LuAC tumor angiogenesis *in vitro*, we first performed an endothelial tube formation assay. In the experiments, human umbilical vein endothelial cells (HUVECs) were infected with lentiviruses carrying Par3 shRNA or control shRNA. It was noted that Par3-depleted HUVECs formed significantly more tubes than were observed in the control groups (Figures 4B, *P* < 0.01). Next, to explore the role of Par3 in tumor angiogenesis *in vivo*, infected A549 and H1299 cells were subcutaneously injected into nude mice. The expression of CD31, a marker of angiogenesis, was more intense in the shPar3 group (Figures 4C, *P* < 0.01).

### Effects of loss of Par3 on invasion, metastasis, and angiogenesis of LuAC are mediated by 14-3-3ζ

**Figure 5:**
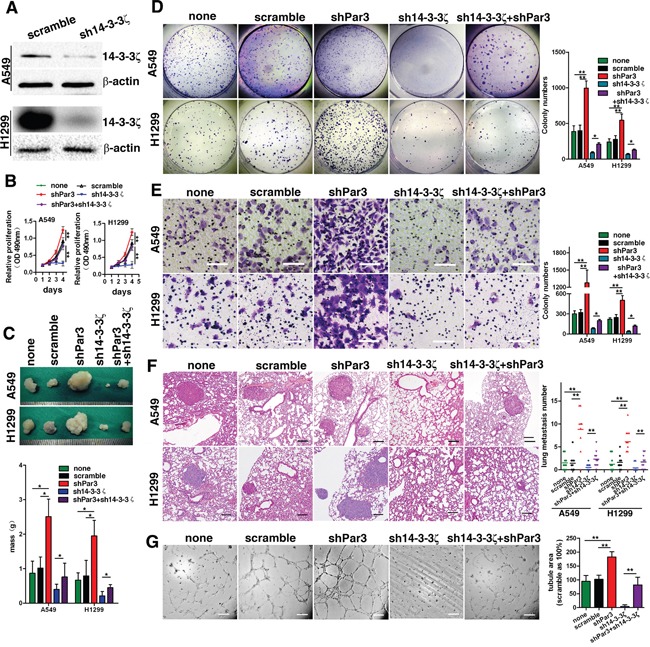
The effects of loss of Par3 on invasion, metastasis, and angiogenesis of LuAC cells are mediated by 14-3-3ζ **A.** Validation of shRNA against 14-3-3ζ in A549 cells by Western blotting. **B.** Growth curves was performed by MTT assay. The results are expressed as the mean±standard deviation (SD) of five independent experiments. **C.** Images of the xenografted tumors formed in nude mice injected with infected A549 and H1299 cells. **D.** Downregulation of 14-3-3ζ abolished the effects of loss of Par3 in promoting A549 and H1299 cell colony formation. **E.** A Transwell invasion assay was performed as described in the Methods. Bars: 100 μm. (magnification, 200×) **F.** Experimental metastasis assays with cells injected in the spleen; representative images are shown for the lung. There were 10 mice in each group. Bars: 100 μm. **G.** The tube formation assay was performed as described in the Methods. Bars: 100 μm. * *P* < 0.05, ** *P* < 0.01.

14-3-3 proteins have been previously shown to activate multiple cellular processes via a variety of different mechanisms [14–17]. The 14-3-3 isofom, 14-3-3ζ protein, is required for the asymmetric localization of Par3 during the polarization of cells. Disruption of the binding between 14-3-3ζ and Par3 results in a loss in epithelial cell polarity [10], suggesting that 14-3-3ζ might play a regulatory role in the function of Par3. To test this hypothesis, we inhibited 14-3-3ζ expression by expressing 14-3-3ζ shRNA in tumor cells (Figures 5A). 14-3-3ζ shRNA abolished the effects of loss of Par3 on promoting A549 and H1299 cell growth (Figures 5B) colony formation (Figures 5D) and subcutaneous tumor growth (Figures 5C). Consistently, when 14-3-3ζ was knocked down, knock-down of Par3 expression could not further promote the invasion (Figures 5E) or metastasis (Figures 5F) of A549 and H1299 cells. Moreover, when 14-3-3ζ shRNA was expressed, loss of Par3 in HUVECs did not increase tube formation (Figures 5G), nor did Par3 knock-down increase the expression of CD31 in xenograft tumors (Supporting Figures 2).

### Loss of Par3 activates Tiam1-Rac signalling via 14-3-3ζ

**Figure 6:**
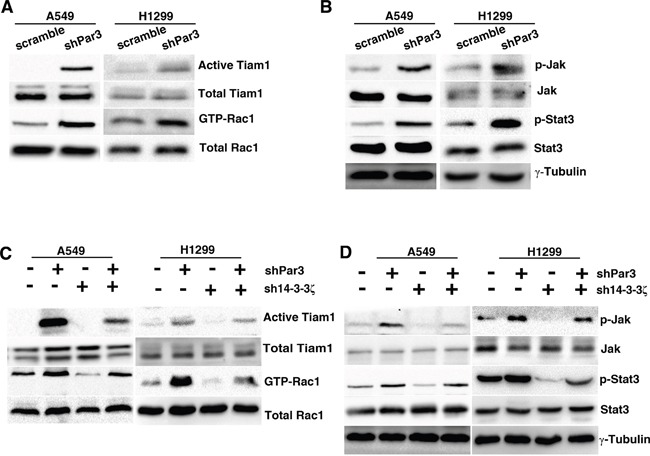
Loss of Par3 activates Tiam1-Rac signaling via 14-3-3ζ **A.** Cell lysates from scramble- or Par3-shRNA A549 cells were subjected to a Tiam1 activity assay and Rac activity assay. The levels of active Tiam1 pulled down by Rac1 G15A agarose beads and total Tiam1 were determined by immunoblotting with Tiam1 antibody. The levels of active Rac1 pulled down by PAK-PBD and total Rac1 were monitored. **B.** Phospho-antibodies were used to detect pJAK^Y1007/8^ and pStat3^Y705^ in control or shPar3-infected cells. **C.** A549 cells were infected with control or Par3 shRNA or 14-3-3ζ shRNA. Cell lysates were subjected to the Tiam1 activity assay and Rac activity assay. **D.** Detection of pJAK^Y1007/8^ and pStat3^Y705^ in each group.

Par3 is known to inhibit Rac activation through binding with Tiam1. We used Rac1 G15A, a Rac1 mutant that binds with active Tiam1, and PAK-PBD pulldown assays to monitor the changes in Tiam1 activation and Rac1-GTP levels in shPar3-infected cells. Par3-knockdown cells showed a marked increase in the levels of active Tiam1 and high Rac1-GTP levels when compared with the control cells (Figures 6A).

As Rac1 can induce JAK-STAT activation in some cells [18], we next asked whether loss of Par3 might result in JAK-STAT activation. We confirmed that knockdown of Par3 caused a marked increase in pJAK^Y1007/8^ and pStat3 ^Y705^ levels as determined by western blotting (Figures 6B). To determine the role of 14-3-3ζ in loss of Par3-induced Tiam1-Rac1 and JAK-STAT activation, we used shRNA to inhibit the expression of 14-3-3ζ in tumor cells. 14-3-3ζ shRNA abolished the effects of loss of Par3 on promoting Tiam1-Rac1 (Figures 6C) and JAK-STAT activation in A549 and H1299 cells (Figures 6D). These results demonstrate that 14-3-3ζ protein is necessary for the loss of Par3 to result in Tiam1-Rac1 and JAK-STAT activation.

### Par3 binds with 14-3-3ζ and loss of Par3 promotes interaction between 14-3-3ζ and Tiam1

**Figure 7:**
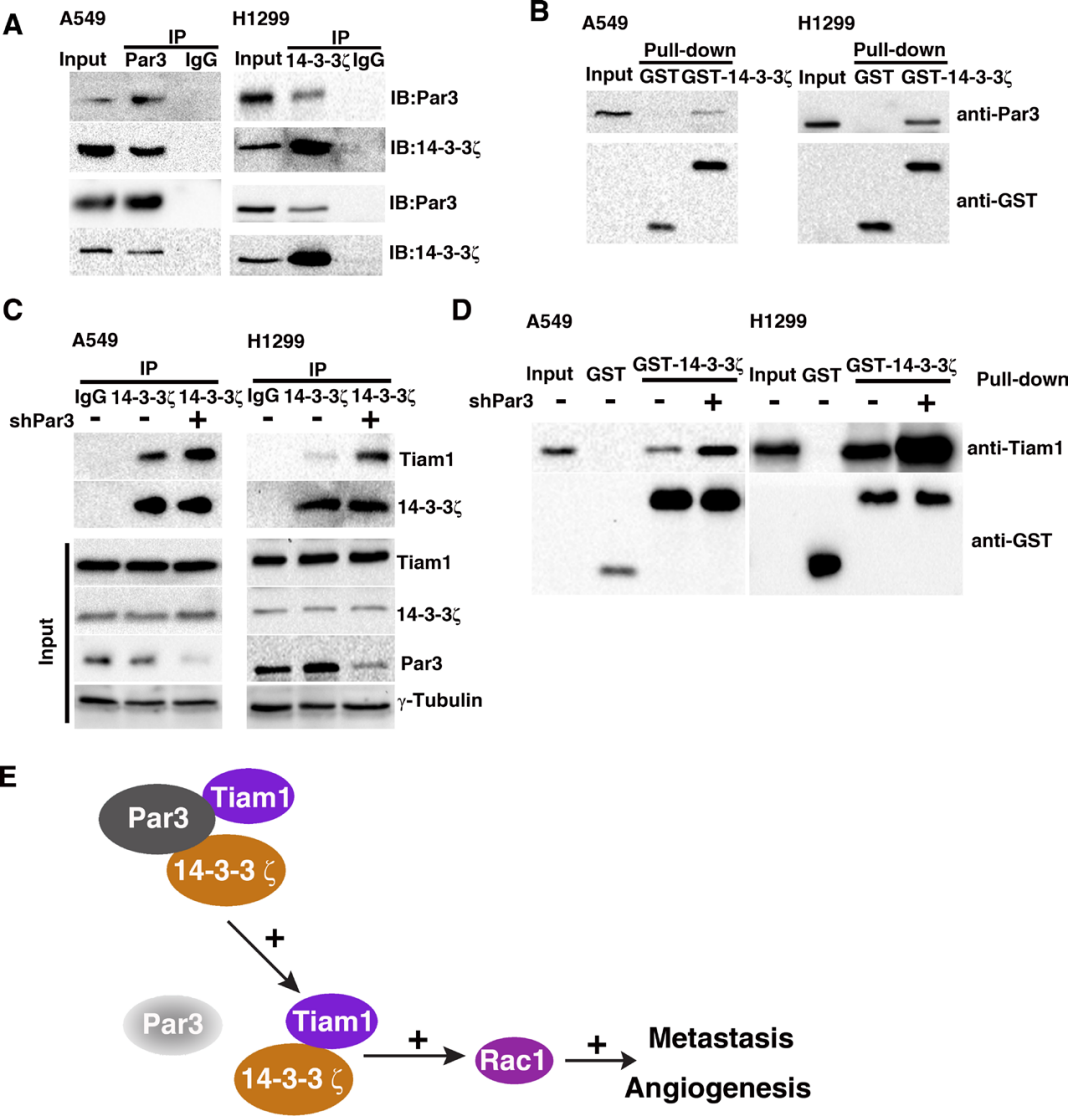
Par3 binds with 14-3-3ζ and loss of Par3 promotes the interaction between 14-3-3ζ and Tiam1 Validation of the binding between Par3 and 14-3-3ζ in A549 cells by immunoprecipitation (**A**) and GST pulldown assay (**B**). The lack of positive bands for the negative control (IgG) demonstrated that the cells did not contain any proteins that could interfere with the interpretation of the results. **C.** A549 cells were infected with control or Par3 shRNA and lysed, and the protein complexes were precipitated with anti-14-3-3ζ antibody. The immunoprecipitates were subjected to Western blotting. **D.** Lysates of A549 cells were incubated with purified GST or GST-14-3-3ζ in the absence or presence of Par3 shRNA. The bound proteins precipitated with glutathione-Sepharose 4B were subjected to Western blotting. The data are representative of at least three independent experiments.

Western blot were performed to investigate whether Par3 affect the expression of 14-3-3ζ. We demostrated that knockdown of Par3 do not affect the protein expression level of 14-3-3ζ(supplement figure 3). To elucidate the role of 14-3-3ζ in the activation of Tiam1-Rac signaling by loss of Par3, we tried to identify whether Par3 could bind to 14-3-3ζ. Immunoprecipitation (Figures 7A) and GST pulldown assay (Figures 7B) assays were performed to investigate the molecular interactions between Par3 and 14-3-3ζ in A549 and H1299 cells and to demonstrate that Par3 could bind directly to 14-3-3ζ. These results suggest that inhibition of Rac1 activation may be caused by the binding of Par3 to 14-3-3ζ.

Next, we examined the mechanism underlying the inhibition of Rac1 activation caused by the binding of Par3 to 14-3-3ζ. Previous studies have shown that 14-3-3 protein overexpression results in the activation of Tiam1 and Rac1 and mediates cell migration [19, 20]. We hypothesized that loss of Par3 could binding with 14-3-3ζ to promotes the interaction between 14-3-3ζ and Tiam1. Immunoprecipitation (Figures 7C) and a GST pulldown assay (Figures 7D) was performed to investigate the changes in binding of 14-3-3ζ and Tiam1. The results demonstrated that the binding of 14-3-3ζ with Tiam1 was enhanced by the loss of Par3. Therefore, it is likely that a conformational change in Tiam1 caused by binding with 14-3-3ζ activates the GEF property of Tiam1 and is promoted by loss of Par3. These results demonstrate that loss of Par3 promotes 14-3-3ζ and Tiam1 interaction to facilitate Rac1 activation and stimulate cancer cell metastasis.

## DISCUSSION

In recent years, multiple studies have shown that Par3 is frequently downregulated in several types of human cancer, including esophageal squamous cell carcinoma [12], breast cancer [8, 11], pancreatic cancer [21], and lung squamous cell carcinomas [22]. However, other reports have shown that Par3 may function as an oncogene in many cancers, including squamous cell carcinomas [23] and papillomas [12]. Loss of Par3 can trigger both apoptosis and proliferation in mammary epithelial cells [24]. It is unknown whether Par3 has a suppressive or oncogenic function in LuAC. In this report, we identified Par3 as a novel, tumor suppressive gene in LuAC.

Loss of Par3 was significantly associated with increased lymph node metastasis (*P* = 0.01) and a poor disease-free survival rate (*P* = 0.01). Our functional studies demonstrated that loss of Par3 promoted cell growth, cell invasion, and tumor formation in nude mice. The effect of loss of Par3 on promoting tumor metastasis was further validated *in vivo*. Because angiogenesis also plays an important role in tumor invasion and metastasis during tumor progression, we tested whether loss of Par3 promotes angiogenesis. As expected, downregulation of Par3 led to increased tube formation by HUVECs and increased angiogenesis in a xenograft tumor model.

Par3 is known to bind with Tiam1 and inhibit its guanine nucleotide exchange activity towards Rac GTPase [25]. However, the underlying mechanisms through which Par3 silencing triggers Tiam1 activation and rapid tumor metastasis are not fully understood. We propose the following model (Figure 7E). First, Par3 binds with 14-3-3ζ and restricts 14-3-3ζ binding to Tiam1. Loss of Par3 promotes the binding of 14-3-3ζ to Tiam1, which triggers the high basal levels of Rac-GTP. Activated Rac1 in turn triggers JAK-STAT pathway activation, resulting in rapid tumor growth and metastasis. A previous study showed that 14-3-3ζ functions as a cofactor with Par3 to establish epithelial polarization [10, 26]. Therefore, we speculated that the downregulation of 14-3-3ζ would enhance the metastasis mediated by loss of Par3. However, contrary to our speculation, we observed decreased metastasis in 14-3-3ζ-depleted cells, indicating that 14-3-3ζ regulating loss of Par3 mediated metastasis by a aPKC-independent mechanism. Instead, we detected that 14-3-3ζ–mediated loss of Par3 caused metastasis in a Tiam1-dependent manner.

14-3-3ζ belongs to the 14-3-3 protein family, which is a class of highly conserved proteins encoded by seven mammalian genes (β, γ, ε, σ, ζ, τ, and η) [27]. Increased expression of 14-3-3ζ has been observed in several human tumors, including human squamous carcinoma, hepatocellular carcinoma, stomach cancer, breast cancer, and several types of lung carcinoma, suggesting that 14-3-3ζ plays an important role in the development and progression of cancer [28]. 14-3-3 proteins are known to occur as homo- or heterodimers in cells and hence can serve as bridging factors. By co-immunoprecipitation, 14-3-3 proteins and Par complexes shown that MARK▪Par1 complexes interact mainly with 14-3-3η, whereas Par3 binds mainly with 14-3-3ζ [29]. However, we cannot exclude the possibility that other members of the 14-3-3 family are also associated with the inhibition of Rac1 activation caused by loss of Par3. Binding of 14-3-3 to Par3 blocks the ability of aPKC to associate with Par3 [26], suggesting that 14-3-3 may affect aPKC activity. An important question for future studies is whether 14-3-3ζ is involved in the loss of Par3–mediated aPKC activation and whether other members of the 14-3-3 family are involved in metastasis induced by Par3 inhibition.

In conclusion, this study demonstrates that loss of Par3 can significantly promote LuAC cell proliferation, invasion, angiogenesis, and metastasis by enhancing 14-3-3ζ binding to Tiam1. The newly identified Par3/14-3-3ζ/Tiam1 pathway helps us to further elucidate the intricate molecular mechanisms of Par complexes and represents a novel strategy for prognosing and treating patients with LuAC.

## MATERIALS AND METHODS

### Cell lines, clinical samples and antibodies

A549, H460 (American Type Culture Collection, USA), H1299 and H23 cells (kindly provided by Dr Jin Yang, Huazhong University of Science and Technology) were grown in Dulbecco's modified Eagle's medium (Sigma-Aldrich, USA) supplemented with 10% fetal bovine serum (Gibco, USA). Human umbilical vein endothelial cells (HUVEC) were isolated from umbilical veins of fresh cords. The cells were maintained in Endothelial Cell Medium (Sciencell, USA). A total of 61 paired specimens (adjacent tissues and tumor) were collected immediately following lobectomy of LuAC patients at the Union Hospital (Wuhan, China). Samples used in this study were approved by the Committees for Ethical Review of Research at Huazhong University of Science and Technology. Par3 (ab64646), Rac1 (ab155938) and CD31 (ab28364) antibodies was purchased from Abcam (Abcam, USA); 14-3-3ζ (sc-1019), anti-pJAK2^Y1007/8^ (sc-21870), anti-JAK2 (sc-278), anti-pSTAT3^Y705^ (sc-7993), anti-ZO-1 (sc-10804), and anti-STAT3 (sc-482) were from Santa Cruz Biotechnology (Santa Cruz, USA); and anti-Tiam1 (STA-422) was from the Active Rac-GEF assay kit (Cell Biolabs, USA).

### Establishment of knockdown cells

Lentiviruses containing shRNAs targeting Par3 and 14-3-3ζ were purchased from Shanghai GeneChem (GeneChem, China) and used to infect cells. The shPar3 target sequence was 5′-GCCATCGACAAATCTTATGAT-3′ and the sh14-3-3ζ target sequence was 5′-GCAATTACTGAGAGACAACTT-3′. Cells infected with a non-effective scrambled shRNA with vector were used as controls. Puromycin (2 μg/mL) was used to select stable clones. After treated with puromycin 2 weeks, multiple single colonies were used in the following experiments.

### DNA constructs

Human cDNA for 14-3-3ζ was amplified from A549 and H1299 cell cDNA and cloned into pcDNA3 (Invitrogen, USA). All of the constructs were cloned into pGEX-2T (GE Healthcare, USA) to prepare GST fusion proteins in bacteria.

### Immunohistochemical and immunofluorescence staining analysis

A549 and H1299 cells growing on glass coverslips were fixed (4% paraformaldehyde), permeabilized (0.2% Triton-X100), and incubated in blocking buffer (5% bovine serum albumin) for 30 min. Primary antibodies (rabbit Par3, 1:300) were detected with appropriate secondary antibodies. Immunohistochemistry was performed as previously described [30]. Briefly, formalin-fixed, paraffin-embedded specimens were deparaffinized, rehydrated, and blocked by 10% normal goat serum. The slides were then incubated with rabbit antibody against Par3 at a dilution of 1:200 and subsequently incubated with biotin-conjugated goat anti-rabbit antibody (Proteintech, China) at a concentration of 1:200 for 30 minutes at 37°C. The immunohistochemical scoring system considering the staining intensity and area extent was applied. The scores were defined according to the cell staining intensity (0 = negative; 1 = weak; 2 = moderate; and 3 = strong) multiplied by the extent of stained cells (0% = 0, 1–24% = 1, 25–49% = 2, 50–74% = 3, 75–100% = 4), leading to scores from 0–12 [31]. To assess tumor angiogenesis, IHC and IF staining were performed to visualize the vascular endothelium using CD31 antibody. The fluorescence was visualized with a laser confocal microscope (FV500; Olympus, Tokyo, Japan).

### Cell migration and invasion assays

Scratch wound migration assays were performed on A549 and H1299 cells as previously described [32]. Cells were cultured on a 35-mm dish until confluence and then wounded using a 10-μl pipette tip. Migration photos were captured at 0 and 24 h after scratching. An invasion assay was performed with growth factor-reduced Matrigel invasion chambers (BD Biosciences, USA) following the manufacturer's instructions. Cells were incubated for 24 h and fixed and counted under a microscope.

### Hanging-drop assay

Drops (20 μl) of cells (2.5×10^5^ cells/ml) were pipetted onto the lid of a 24-well culture plate. The lid was quickly inverted so that the drops were hanging from the lid with the cells suspended within them. To prevent evaporation, serum-free culture medium was placed in the well. After 18 h, the lid of the plate was inverted and photographed using a Nikon Eclipse TS100 microscope (Nikon, Japan). At least 6 drops were analyzed per experiment.

### Colony formation assay and MTT assay

For the colony formation assay, 500 cells were cultured in 35-mm dishes. The medium was changed every 3 days. After 2 weeks, the cells were fixed and stained with crystal violet, and colonies with a diameter exceeding 2 mm were counted. Triplicate experiments were performed. For the MTT (3-(4, 5-dimethylthiazol-2-yl)-2, 5-diphenyltetrazolium bromide) assay, cells were plated at an initial density of 5 × 10^3^/well in 96-well plates and incubated in complete culture medium. After treatment with 20 μl MTT (5 mg/ml, Sigma) and dimethylsulfoxide (200 μl, Sigma), the absorbance was measured at 490 nm with subtraction of the baseline reading.

### Endothelial tube formation assay

The Matrigel solution was thawed at 4°C for 30 min, HUVECs (1 × 105 cells/well) infected with lentiviruses carrying shRNA were added onto solidified Matrigel in 100 μl medium. After 8–18 h of incubation, endothelial cell tube formation was assessed, and tube area was calculated under light microscopy by using Image-Pro Plus software [33].

### Tumor xenograft model

Nude mice (5 weeks old) were purchased from Beijing HFK Bio-Technology Co, LTD. (Beijing, China) for studies approved by the Tongji Medical College Animal Experiments Committee. The mice were maintained in the accredited animal facility of Tongji Medical College. For tumorigenicity detection, cells (5×10^6^ cells/100 μl DMEM) were subcutaneously injected into nude mice. Volumes were determined by caliper measure-ment of the length, width, and height of each tumor. After approximately 6 weeks, the animals were sacrificed, and the subcutaneous tumors were weighed. For experimental metastasis assays, intrasplenic injections of nude mice with infected cells (5×10^4^ cells/50μl DMEM) were performed on exteriorized spleens as previously described [34]. After 5 min, the splenic artery and vein were ligated, and the spleen was excised. After 6 weeks, the mice were killed, and lung tissues were excised and embedded in paraffin for further study.

### Western blotting

Cells were lysed and electrophoresed by sodium dodecyl sulfate (SDS)-polyacrylamide gel electrophoresis (PAGE) and electroblotted onto a PVDF membrane (Millipore). Then the membranes were incubated with primary and secondary antibodies, and the immune complexes were detected with an enhanced chemiluminescent reagent (Thermo).

### RNA extraction and real-time RT-PCR

Reverse transcription reaction and real-time quantitative PCR were performed as described previously [35]. Total RNA was extracted from freshly frozen normal and malignant human LuAC specimens using the TRIZOL reagent (Invitrogen). cDNA was synthesized using the PrimeScript™ RT reagent kit (Takara, Japan). The cDNA was subjected to real-time PCR using the SYBR® Premix Ex Taq™ Kit (Takara, Japan). 18S rRNA was used as an internal control. The sequences of the Par3 primers were as follows: forward, 5′-CAGACAGAACTACTAACTTCGCC-3′; reverse, 5′-ATGCCTCGGATGAAGAGTCCT-3′. The relative expression level of Par3 was normalized to the 18S rRNA reference (ΔCt) and related to the amount of gene in control sample, which was defined as the calibrator at 1.0. Par3 mRNA levels in lung tumors that were higher than the median value (50th) were defined as ‘high’, and levels equal and/or lower than the median value were defined as ‘low’.

### Rac-GTP pulldown assay

Cells were cultured to approximately 80-90% confluence. The active form of Rac1 was pulled down by PAK PBD (p21-binding domain of p21-activated protein kinase) agarose beads. Assays were performed using the small GTPase activation assays kit (STA-401-1, Cell Biolabs) according to the manufacturer's instructions.

### Tiam1-activity assay

Active Tiam1 was pulled down by Rac1 G15A agarose beads. Assays were performed using the active small GTPase active GEF assay kit (STA-422, Cell Biolabs) according to the manufacturer's instructions.

### Immunoprecipitation

Infected A549 and H1299 cells were collected and sonicated in immunoprecipitation (IP) buffer. The cell lysates were centrifugation at 12,000×g for 15 min at 4°C and then incubated with anti-Par3 or anti-14-3-3ζ antibody covalently coupled to protein A/G-agarose beads (Santa Cruz Biotechnology, USA) at 4°C overnight. Before the addition of antibodies, a small portion of each supernatant was preserved for later Western blotting (input). The immunoprecipitates were washed three times with IP buffer. The bound proteins were eluted from beads by boiling in SDS sample buffer for 5 min and subjected to Western blotting.

### GST pulldown assay

The GST fusion proteins were expressed in *Escherichia coli* and purified using glutathione–agarose beads (GST beads, GE Healthcare Life Sciences). The infected cells were scraped from the plate at 4°C. After centrifugation of the cell lysates for 15 min at 15,000 × *g*, the supernatant was collected and then incubated with purified GST or GST-14-3-3ζ and glutathione–agarose beads at 4°C overnight. The bound proteins were eluted in SDS sample buffer and subjected to Western blotting.

### Statistical analysis

Statistical analysis was performed using the EmpowerStats statistical software program Version 2.16.1. The mRNA levels of Par3 in LuAC tumor tissues and adjacent nontumor tissues were compared using a paired Student t test. Clinical correlations were analyzed by χ^2^ test, and survival analyses were assessed using Kaplan–Meier plots and log-rank tests. Multivariate survival analyses were performed by Cox regression model.

## SUPPLEMENTARY MATERIALS FIGURES


