# Supplementary material for: Loss of Par3 promotes lung adenocarcinoma metastasis through 14-3-3ζ protein
Source: Oncotarget. 2016 Aug 31;7(39):64260–73. doi: 10.18632/oncotarget.11728 (PMC5325440; doi:10.18632/oncotarget.11728)
Supplement: Supplementary file 1 [file oncotarget-07-64260-s001.pdf]

Loss of Par3 promotes lung adenocarcinoma metastasis through 14-3-3ζ protein

SUPPLEMENTARY FIGURES

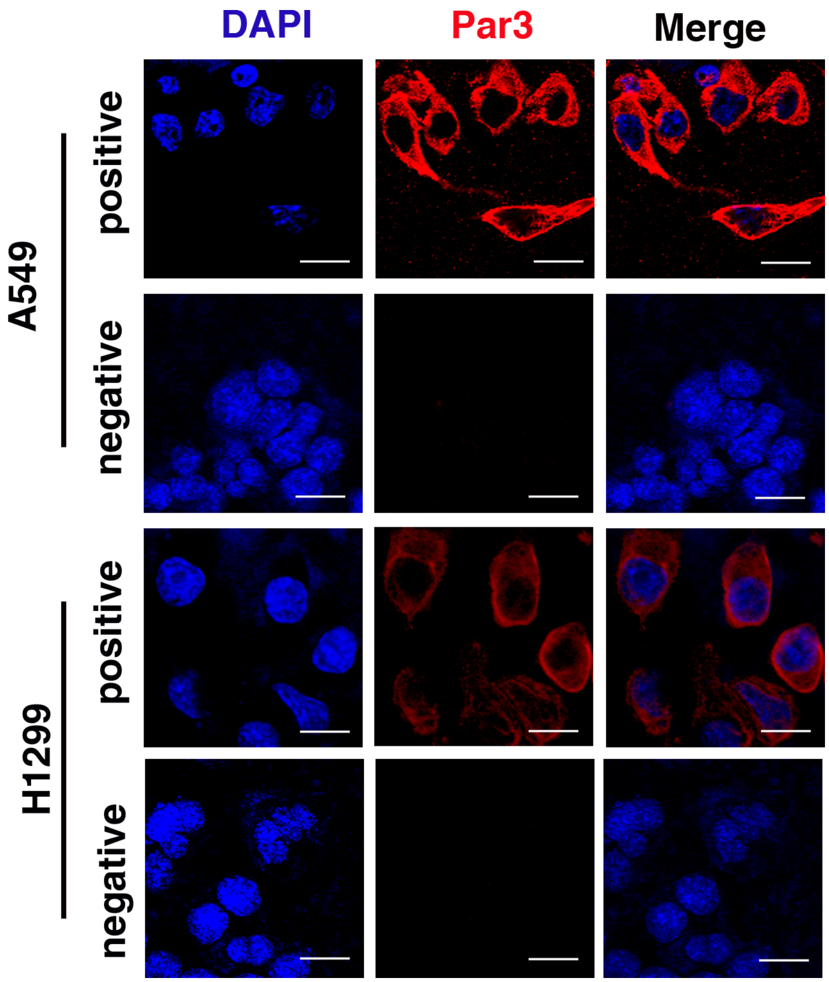

Supplementary Figure 1: Validation of negative control in A549 and H1299 cells by immunofluorescent staining. Negative group incubated with no primary antibody but with a goat anti-rabbit secondary antibody. Positive group incubated with Par3 antibody. Bars: 25 μm.

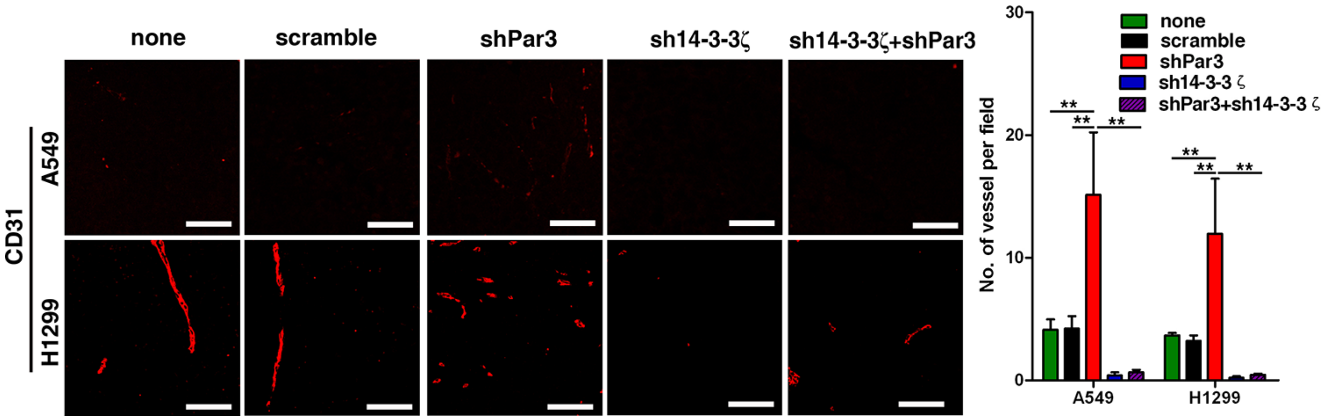

Supplementary Figure 2: Tumor angiogenesis was assessed with IF staining using an antibody against CD31. Bars: 25 μm. All quantitative data are shown in the right panel. * $P < 0.05$, ** $P < 0.01$.

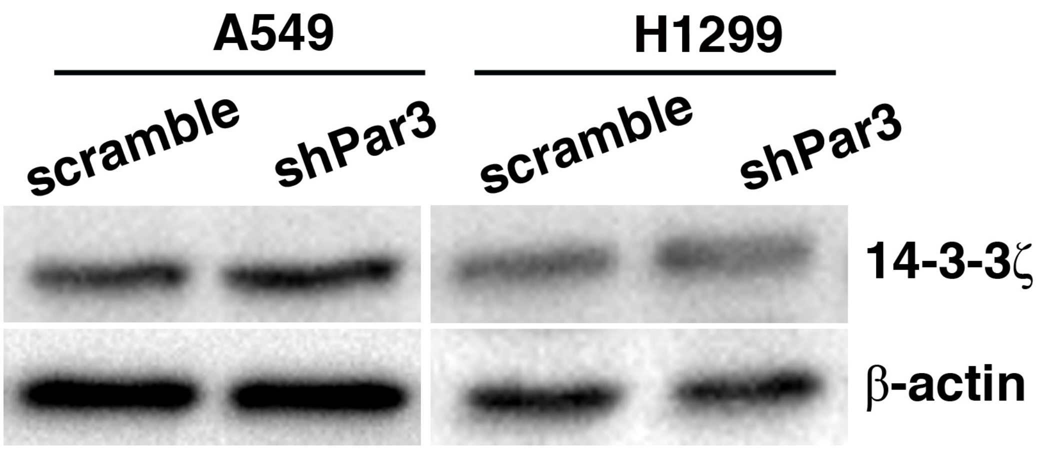

Supplementary Figure 3: Western blot analysis of 14-3-3ζ expression in A549 and H1299 cells.
